# Surface nitridation of Li_4_Ti_5_O_12_ by thermal decomposition of urea to improve quick charging capability of lithium ion batteries

**DOI:** 10.1038/s41598-021-92550-z

**Published:** 2021-06-22

**Authors:** Jihyun Jang, Tae Hun Kim, Ji Heon Ryu

**Affiliations:** 1 Department of Chemical and Biological Engineering, Seoul National University, 1 Gwanak-ro, Gwanak-gu, Seoul, 08826 Republic of Korea; 2 Department of Chemical Engineering and Biotechnology, Korea Polytechnic University, 237 Sangidaehak-ro, Siheung-si, Gyeonggi-do 15073 Republic of Korea; 3 Graduate School of Knowledge-Based Technology and Energy, Korea Polytechnic University, 237 Sangidaehak-ro, Siheung-si, Gyeonggi-do 15073 Republic of Korea

**Keywords:** Energy science and technology, Materials science

## Abstract

As the application of lithium-ion batteries in electric vehicles increases, the demand for improved charging characteristics of batteries is also increasing. Lithium titanium oxide (Li_4_Ti_5_O_12_, LTO) is a negative electrode material with high rate characteristics, but further improvement in rate characteristics is needed for achieving the quick-charging performance required by electric vehicle markets. In this study, the surface of LTO was coated with a titanium nitride (TiN) layer using urea and an autogenic reactor, and electrochemical performance was improved (initial Coulombic efficiency and the rate capability were improved from 95.6 to 4.4% for pristine LTO to 98.5% and 53.3% for urea-assisted TiN-coated LTO, respectively. We developed a process for commercial production of surface coatings using eco-friendly material to further enhance the charging performance of LTO owing to high electronic conductivity of TiN.

## Introduction

Because lithium-ion batteries (LIBs) exhibit a higher capacity and superior power characteristics compared to other conventional secondary batteries, most energy storage and conversion systems used in electronic devices are being replaced by LIBs. However, to expand the range of applications to power tools and electric vehicles (EVs), the energy and power density of current LIBs must be improved^1–3^. Several methods have been developed to reduce the resistance by replacing the cell components and design parameters for power characteristic enhancements of batteries, but the most effective solution is to use an active material with an excellent rate capability^4,5^.

One of the main applications of LIBs is powering EVs. The charging speed of vehicles is a major concern for the commercialization of LIBs. In terms of material, the meaning of improving the charging ability is fast lithium ion extraction from the positive electrode and insertion into the negative electrode; the latter reaction is known as the rate-determining step^6,7^. Several studies have sought to enhance the rate performance of negative electrode materials^8–10^. Specifically, there has been considerable interest in lithium titanium oxide (Li_4_Ti_5_O_12_, LTO), which is a series of titanium oxides that are representative of a negative electrode material with a good rate capability^11–14^. Three main reactions enable the high-power characteristics of LTO: (1) lithium-ion intercalation and de-intercalation reactions occur almost without changes in the 3D structure of LTO (< 0.1–0.2%), (2) the reaction voltage of this reaction is a relatively high, ~ 1.55 V (vs. Li/Li^+^), preventing the reduction in electrolytes and the formation of solid electrolyte interphase (SEI), and (3) nano-sized LTO particles can be used because there is almost no irreversible capacity loss from this electrolyte reduction^15–22^. However, rate capability must be improved further for high-power and quick-charging applications.

Improving the rate characteristics of LTO through surface treatments is a prominent approach employed by many studies because the electronic conductivity is extremely low (< 10^−13^ S cm^−1^)^23,24^. A study has shown that coating the LTO surface with highly electric-conductive Ti–N via heat treatment at a high temperature using NH_3_ gas improves the cell performance (electronic conductivity of Ti–N: 4000–55,000 S cm^−1^)^25–27^. However, owing to the toxicity of NH_3_ gas, this method cannot be used for mass production. In order to address this drawback, an autogenic reactor was used to develop a new synthesis method for the surface coating of LTO using urea (NH_2_CONH_2_) as a coating material instead of toxic NH_3_. Urea is known to decompose below 200 °C and generate NH_3_ gas^28^, so it can be used as a coating material for an autogenic reactor-based treatment that performs nitridation at temperatures above 700 °C^25^. Since the surface coating was performed by gas-phase reaction, all surface of LTO particles can be covered uniformly, and this method is simple and applicable to mass production. In this study, the urea-treated LTO demonstrated a uniform coating layer without changes to its crystal structure as well as improved power characteristics compared to conventional materials.

## Results and discussion

**Figure 1:**
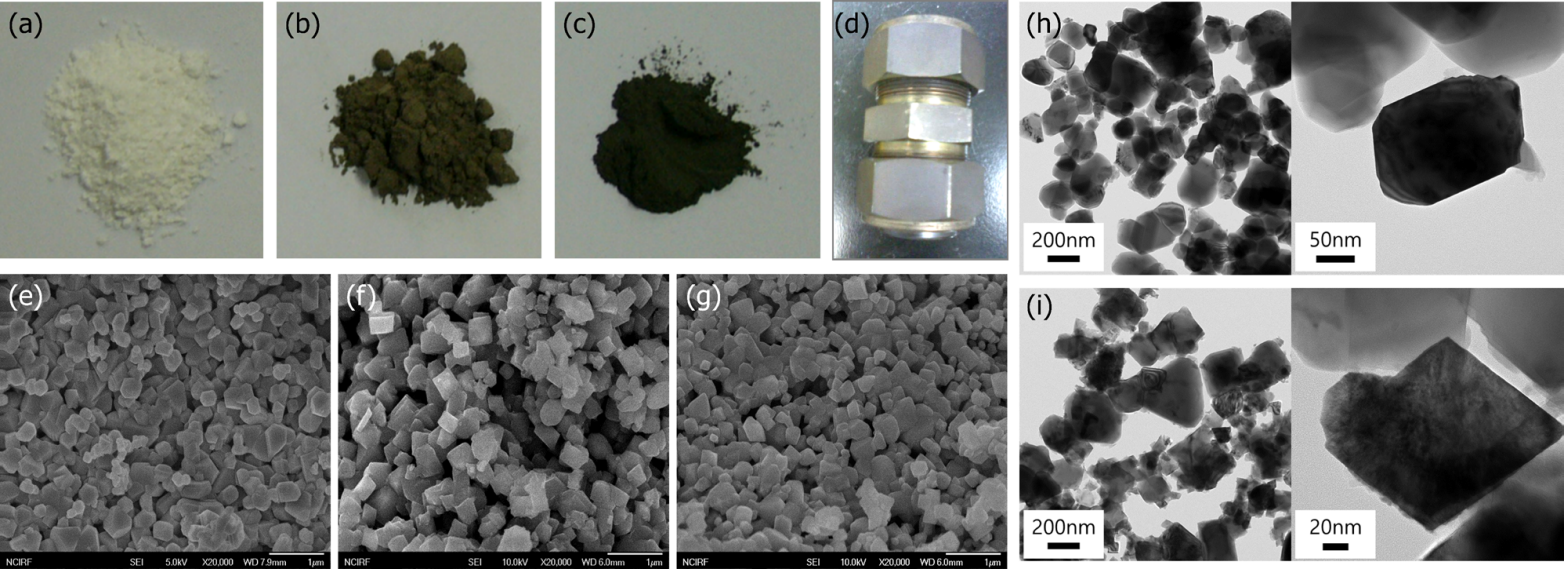
Powder color and field emission scanning electron microscopy images of (**a**, **e**) pristine LTO and surface-coated LTO using urea contents of (**b**, **f**) 10 wt% and (**c**, **g**) 20 wt%, respectively. (**d**) Autogenic reactor used in the coating of the LTO surface. Transmission electron microscopy images of (**h**) pristine LTO and (**i**) 10 wt% urea-assisted coated LTO.

Figure 1 shows the changes in powder colors and particle shapes according to urea coating through the powder images and FE-SEM images. When the amount of urea used in the surface coating increased, the powder color became darker, indicating that the darkness of color is related to the thickness of the coating layer on the surface of LTO (Fig. 1a–c). Figure 1e–g demonstrates that there is no significant difference in particle size, although the shapes of the particles change into a more cubic form after treatment. The shapes of the particles expectedly became more compact due to high pressure and temperature conditions when a coating was performed in the autogenic reactor. TEM analysis confirmed that a new phase with a slight roughness was formed on the surface of the particles in the surface-coated LTO using 10 wt% urea content, which differed from the clean surface for a pristine LTO (Fig. 1h,i). The low-magnitude images in Fig. 1i indicate that this new phase was well-attached to the surfaces of particles.

**Figure 2:**
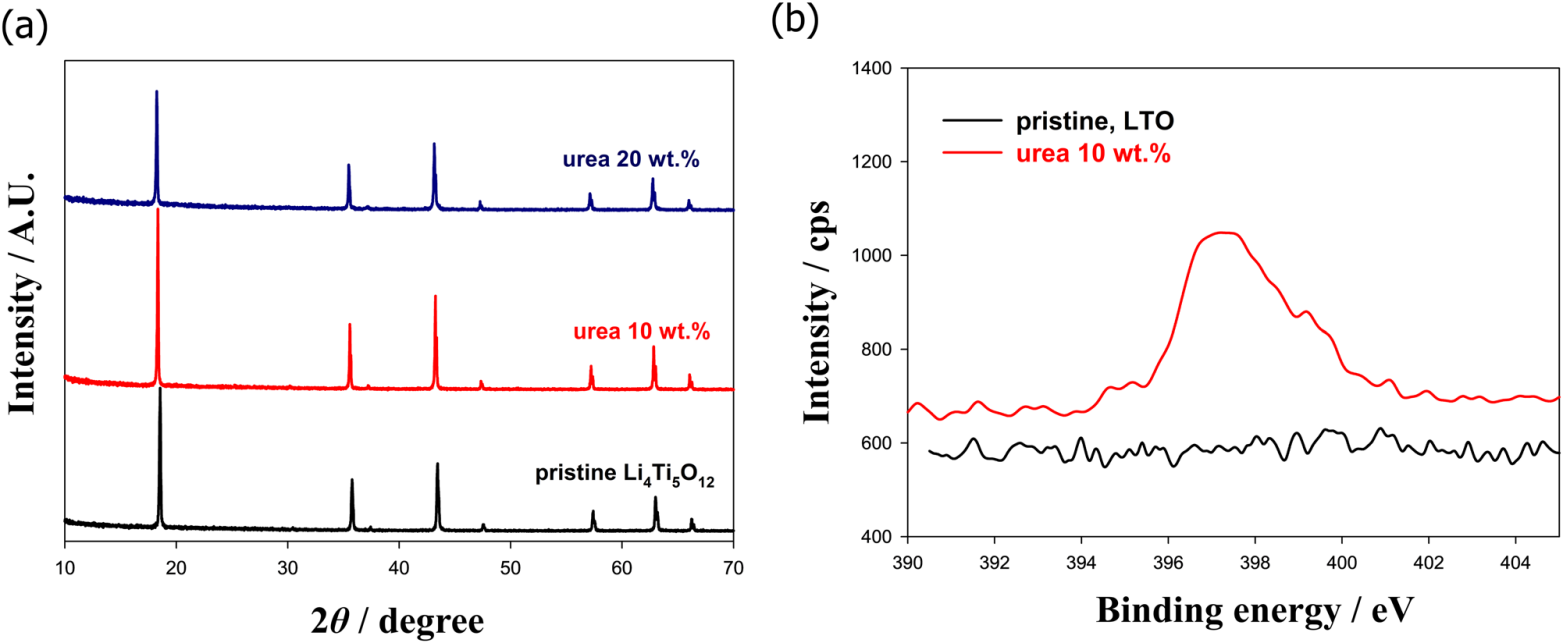
(**a**) XRD patterns of pristine LTO and surface-coated LTO after adding urea contents of 10 wt% and 20 wt%. (**b**) N *1s* XPS results of pristine LTO and 10 wt% urea-assisted LTO.

Because the powder color and particle shape changed after surface treatment with urea, the crystal structures of these materials were confirmed. Figure 2a demonstrates that there was no difference in the bulk structure even after coating using the autogenic reactor, which means that this synthesis method can solely cover the surface with a new material without affecting the crystal structure of the mother phase. Surface analysis of the surface-treated LTO synthesized urea-assisted coating method was performed via the XPS and HR-TEM analyses. N *1s* XPS result (Fig. 2b) shows that the identity of the coating material on the surface of LTO is a nitride-based material. Generally, an autogenic reactor-based surface coating enables the consistent presence of NH_3_ gas generated via the decomposition of urea at high temperatures, and a highly concentrated NH_3_ atmosphere near the LTO can also be generated^29^. In the previous studies, the binding energy of Ti−N bond was 396.5 eV in LTO and TiO_2-x_N_y_ materials, which is well-matched with our result and it can be confirmed that N replaced at the O site on the surface of LTO resulting Ti−N layer was created^25,30,31^. Note that there is no N−O and C=N bonds at above 400 eV mentioned in previous research^32^. The change of powder color from white to black can be also an indirect evidence that the surface of LTO was covered to highly-conductive layer (Fig. 1b,c). The characteristic of the highly-conductive layer was also confirmed by the measurement of electric conductivity. TiN-coated LTO showed the electrical conductivity of 4.89 × 10^−7^ S cm^−1^ and 3.04 × 10^−6^ S cm^−1^ for 10 wt% and 20 wt% urea-assisted LTO, respectively, which is much higher than pristine LTO (4.34 × 10^−8^ S cm^−1^)^35^.

**Figure 3:**
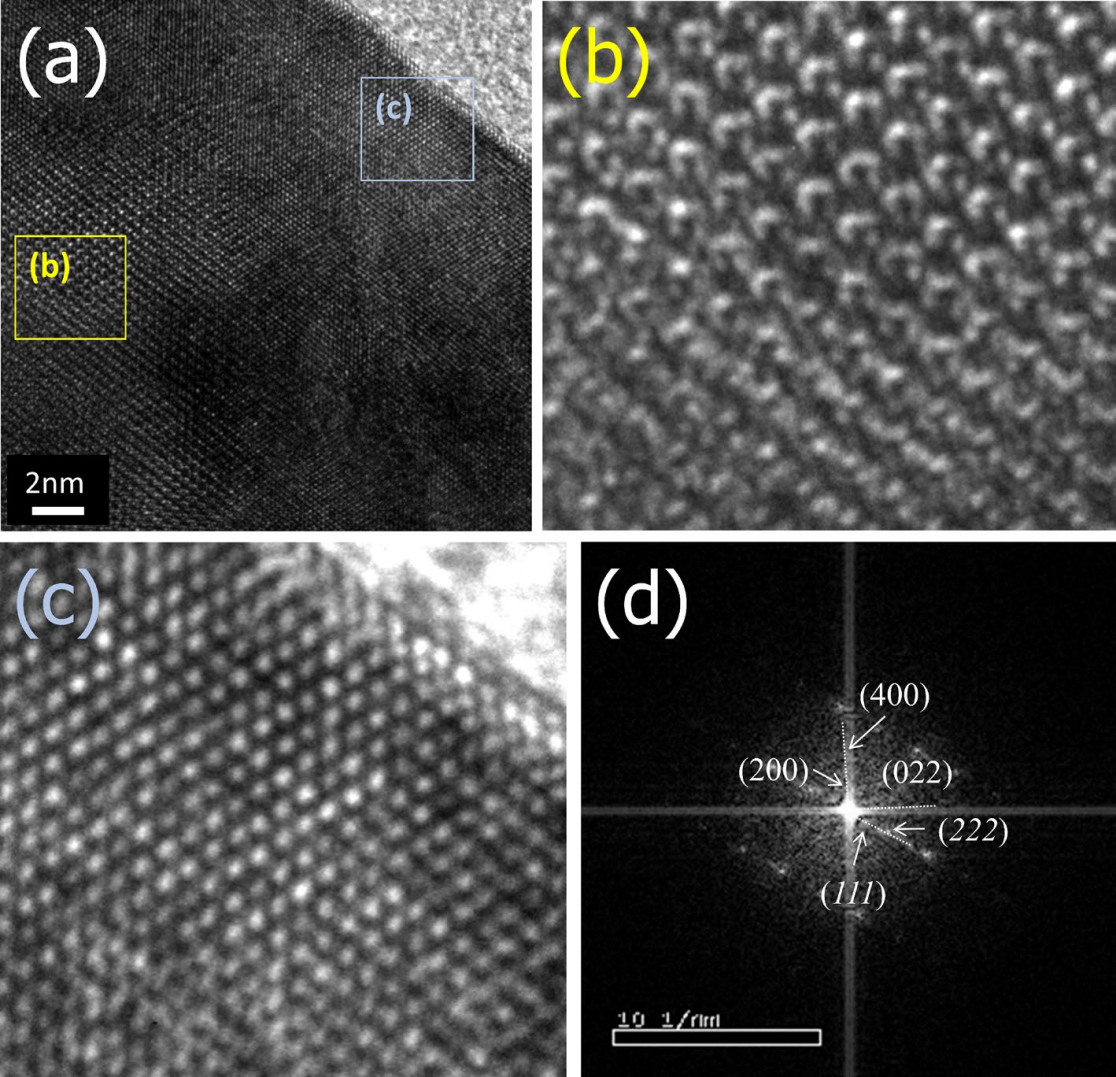
(**a**) High resolution transmission electron microscopy images of surface-coated LTO by adding 10 wt% urea. (**b**), (**c**) Enlarged images for (**b**) and (**c**) boxes in (**a**). (**d**) FFT image for (**c**).

In addition, HR-TEM demonstrates that the coating layer is uniform, with a thickness of approximately 3–4 nm (Fig. 3a). As can be seen in the FFT image of the coating layer (Fig. 3d), the surface of LTO (Fig. 3c) has a cubic crystal structure, which is different from a spinel structure of bulk LTO (Fig. 3b)^33,34^. In the active material coating, a thin thickness and uniformity are highly important because they are directly related to the power performance of the electrode. If there is a difference in thickness of the coating layer according to each position on the surface of the active material when charging and discharging, non-uniform current distribution may occur; accordingly, lithium insertion and extraction will proceed unevenly in the active material particles. As the C rate increases, the non-uniformity of state of charge in the particle increases and eventually acts as a large resistance, which is the main cause of lowering the rate capability of the cell. A uniform and thin Ti–N layer coated through a gas-phase reaction performed in closed reactor enables to improve the power performance of LTO electrode.

**Figure 4:**
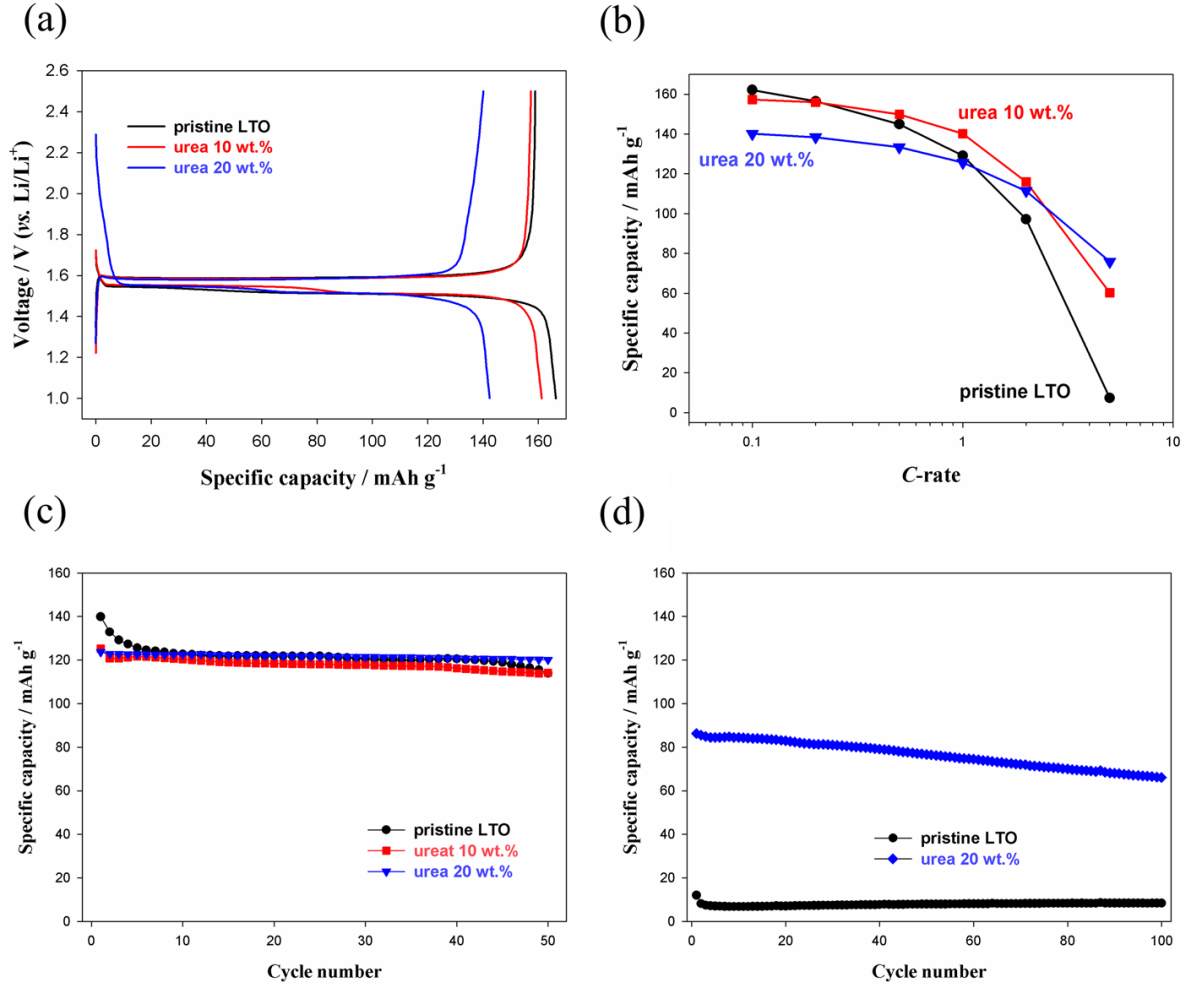
(**a**) First cycle voltage profile, (**b**) quick-charge performance, and cycle performance at (**c**) 1 C and (**d**) 5 C of pristine and urea-assisted LTO. Voltage profile was obtained at a C rate of 0.1 C, and rate capability was examined at C rates of 0.1, 0.2, 0.5, 1, 2, and 5 C.

Figure 4 shows the first-cycle galvanostatic voltage profiles and power performances of pristine LTO and urea-assisted TiN-coated LTO. The first-cycle charge and discharge capacities were 166.3 mAh g^−1^, 158.9 mAh g^−1^ for pristine LTO, 161.2 mAh g^−1^, 157.3 mAh g^−1^ for 10 wt% urea-assisted TiN-coated LTO, and 142.3 mAh g^−1^, 140.1 mAh g^−1^ for 20 wt% urea-assisted TiN-coated LTO, respectively (Fig. 4a and Table 1). It demonstrates that in the case of LTO coated with 10 wt% urea, the irreversible capacity is smaller during the first-cycle charging than that of the pristine counterpart, and thus the initial Coulombic efficiency (ICE) is improved from 95.6 to 97.6%. Note that 20 wt% urea-assisted TiN-coated LTO showed ICE of 98.5%. This must have been caused by the thin and uniformly coated surface of TiN-coated LTO. Although side reactions of the electrolyte rarely occurred on the surface of LTO, even a small amount of decomposition reaction was reduced at the surface of the Ti–N layer. This is likely a product of the beneficial passivation characteristics of the Ti–N layer from the viewpoint of the electrolyte reduction reaction. Generally, the layer with high electron conductivity is not a useful protective film in preventing the reduction reaction of the electrolyte. Nevertheless, since Ti–N layer is likely to have larger resistance in the electrolyte reduction and decomposition reaction than Ti–O layer because of the difference in electron density, the ICE can be improved^31,35–37^. However, when the urea content increased to 20 wt%, the capacity decreased. This was caused by the increase in the amount of TiN, which was an irreversible phase. The quick-charging characteristics of the three materials shown in Fig. 4b demonstrated that LTO coated with 20 wt% urea content had the best rate performance, although its initial capacity was low. Discharge capacities at 2 C and 5 C-rates were 97.2 mAh g^−1^, 7.3 mAh g^−1^ for pristine LTO, 115.9 mAh g^−1^, 60.2 mAh g^−1^ for 10 wt% urea-assisted TiN-coated LTO, and 111.4 mAh g^−1^, 75.9 mAh g^−1^ for 20 wt% urea-assisted TiN-coated LTO, respectively. Note that the ratio between low C-rate (0.1 C) and high C-rate (5 C) of pristine and 20 wt% urea-assisted TiN-coated LTO were 4.4% and 53.3%, respectively (Table 1). In addition, this improved rate capability of TiN-coated LTO also can be confirmed by the comparison of cycle performance in high C-rate. As can be seen in Fig. 4c and d, three LTO showed similar cycleability in 1 C, whereas TiN-coated LTO exhibited much improved cycle performance in 5 C than pristine. This means that as the content of the coated TiN layer increased, the electronic conductivity of the LTO-containing electrode was enhanced because the small portion of the TiN-coated LTO surface was reduced to + 3. As mentioned above, since a relatively small electronic conductivity of LTO impedes quick-charging performance (< 10^−13^ S cm^−1^, the white color of LTO powder in Fig. 1a), the introduction of highly-conductive Ti–N layer on the surface of LTO can improve rate characteristics of a battery, especially during charging.

**Table 1:** First-cycle charge and discharge capacities and rate capabilities for pristine LTO and urea-assisted TiN-coated LTO.

	Capacity @ 0.1 C-rate (1st cycle)			Capacity @ 5 C-rate	
	Charge	Discharge	ICE	Discharge	Ratio (0.1 C/5 C)
	mAh g^−1^	mAh g^−1^	%	mAh g^−1^	%
Pristine	166.3	158.9	95.6	7.3	4.4
10 wt% urea-assisted	161.2	157.3	97.6	60.2	37.3
20 wt% urea-assisted	142.3	140.1	98.5	75.9	53.3

## Conclusion

This research has demonstrated that the high-power characteristic of LTO can be further improved through surface modification via the coating of Ti–N layer, which has high electronic conductivity. Even with high lithium ion mobility, because LTO had a relatively low electronic conductivity, the coating of Ti–N layer on the surface improved the quick-charging performance. Specifically, a thin and uniform Ti–N layer was implemented with an autogenic reactor using eco-friendly urea as a coating reagent instead of toxic NH_3_. Due to the uniformity of coated layer, the electrolyte decomposition during the first-charging reaction was reduced and resulted in improved ICE in TiN-coated LTO (95.6% for pristine LTO and 98.5% for 20 wt% urea-assisted TiN-coated LTO). In addition, extremely-high electronic conductivity of Ti–N layer compared with LTO increased rate capability, from 4.4% of pristine LTO to 53.3% of 20 wt% urea-assisted TiN-coated LTO. The surface-coating process which can be applicable directly into commercial production using eco-friendly material was developed and it can enhance the electrochemical performance including rate capability of LTO owing to uniform and highly-conductive Ti–N layer.

## Methods

### Material preparation

Nano-sized LTO was prepared through a solid-state reaction at 800 °C for 3 h after high-energy bead-milling between Li_2_CO_3_ (99.0%, Sigma-Aldrich, USA) and anatase TiO_2_ (99.5%, Sigma-Aldrich, USA). Nano-sized LTO was placed in an autogenic reactor (316 stainless steel, Swagelok) with urea (99.0%, Sigma-Aldrich, USA) in the weight ratio of 5:1, and the cap was closed to isolate the reactor from the outer atmosphere. Coated LTO was obtained by heating the reactor to 700 °C at a heating and cooling rate of 10 °C min^−1^ in an electric box furnace in air. (Note that an autogenic reactor used in this study was closed system).

### Electrode fabrication

The composite electrode was fabricated by mixing the active material, Denka black (conducting agent, Denka, Japan), and polyvinylidene fluoride (binder, KF1300, Kureha, Japan)) with a weight ratio of 95:2:3. The active material and Super P were mixed with a KF1300 solution (6 wt% in N-methyl-2-pyrrolidone) to obtain a slurry. The slurry was coated on the Al foil, and the electrode was dried under vacuum at 120 °C for 12 h. The electrode mass loading was adjusted to about 5.0 mg cm^−2^.

### Electrochemical characterization

The electrochemical performance was evaluated using a two-electrode 2032-type coin cell. The electrolyte was 1.3 M LiPF_6_ dissolved in a mixture of ethylene carbonate and ethyl methyl carbonate (3:7, volume ratio). A porous polypropylene film was used as the separator, and metallic lithium foil was used as a counter electrode in coin cell. All fabrication processes were conducted in a glove-box filled Ar atmosphere. A WBCS-3000 cycler (WonaTech, Korea) was used to perform galvanostatic charge and discharge experiments at a current density of 17.5 mA g^−1^ over a voltage range of 1.0−2.5 V (vs. Li/Li^+^) in a temperature-controlled oven (25 °C). The rate capability experiment was conducted by raising both charging and discharging rates from 0.1 to 0.2 C, 0.5 C, 1 C, 2 C, and 5 C, and each step was cycled in triplicate (1 C = 175 mAh g^−1^).

### Physical analysis

The particle size and morphology of the synthesized powders were examined via field emission scanning electron microscopy (FE-SEM, JEOL JSM-6700F, Japan) and high-resolution transmission electron microscopy (HR-TEM, JEOL JEM-3010, Japan). The XRD patterns were obtained with a D8-Brucker diffractometer (Bruker, Germany) using Cu-K_α_ radiation (1.54056 Å) operated at 40 kV and 40 mA and continuous scanning at a rate of 5° min^−1^. For X-ray photoelectron spectroscopy (XPS, Sigma probe, USA) analysis, Al-K_α_ (1486.6 eV) X-ray radiation produced at a constant power of 100 W (15 kV and 6.67 mA) was illuminated on a spot radius of 200 µm, and the pass energy for the detector was 30 eV. The electrical conductivity was measured by a powder resistivity measurement system (HPRM-M2, HAN TECH).
